# Nux-Vomica^200^ in Labor

**Published:** 1891-02

**Authors:** W. A. Yingling

**Affiliations:** Nonchalanta, Kansas


### NUX-VOMICA$^{200}$ IN LABOR.

W. A. Yingling, M. D., Nonchalanta, Kansas.

* * * The patient who was the victim of the attack of puerperal convulsions reported in The Homœopathic Physician, October number, page 464, has just been safely and easily delivered of a bouncing boy.

She had no trouble to speak of. She had been visiting the day previously and had eaten largely of fat pork and sausage which deranged the stomach. Frequent calls to stool led me to give Nux-vomica$^{200}$ , but it proved useless. I then learned for the first time of the sausage, and gave her Pulsatilla$^{200}$. It failed. The pains continued to delay. Walking the floor, frequent and fruitless calls to stool, told me that Nux-vomica must be the remedy. Yet it did not work. I gave her a cup of hot water to drink, and some time afterward one dose of Nux-vomica$^{200}$. She was compelled to take to the bed at once, with steady effective labor-pains, and in fifteen minutes was easily delivered. Do not forget hot water when the stomach is deranged and the indicated remedy fails to act. It has served well.—[Extract from a letter to the Editors.]

